# Tetralogia de Fallot Associada a Artéria Subclávia Direita Aberrante. Implicações Clínicas

**DOI:** 10.36660/abc.20210880

**Published:** 2022-07-13

**Authors:** Maciej Michałowski, Pawel Tyczynski, Magdalena Lipczynska, Anna Wójcik, Piotr Hoffman, Adam Witkowski, Ilona Michałowska

**Affiliations:** 1 National Institute of Cardiology Department of Interventional Cardiology and Angiology Warsaw Polônia National Institute of Cardiology – Department of Interventional Cardiology and Angiology, Warsaw – Polônia; 2 National Institute of Cardiology Department of Congenital Heart Diseases Warsaw Polônia National Institute of Cardiology – Department of Congenital Heart Diseases, Warsaw – Polônia; 3 National Institute of Cardiology Department of Radiology Warsaw Polônia National Institute of Cardiology – Department of Radiology, Warsaw – Polônia

**Keywords:** Cardiopatias Congênitas, Tetralogia de Fallot, Aorta Torácica, Obstrução Esofágica, Artéria Subclávia

## Abstract

Desde a primeira descrição da tetralogia de Fallot (ToF) em 1671 por Niels Stensen e em 1888 por Étienne-Louis Arthur Fallot, vários trabalhos relataram essa anomalia juntamente com suas variantes e anomalias cardiovasculares concomitantes. A artéria subclávia direita aberrante (ASDA) é a anomalia do arco aórtico mais comum. Diferentemente da artéria subclávia esquerda aberrante, a ocorrência de ASDA em pacientes com ToF só foi relatada casuisticamente. Apresentamos dois pacientes de ToF com ASDA. É importante notar que o conhecimento da coexistência das duas anomalias tem pontos muito práticos durante correções endovasculares ou cirúrgicas de defeitos cardíacos congênitos (inclusive ToF).

## Introdução

As anomalias do arco aórtico podem ser isoladas ou podem estar associadas a outros defeitos cardíacos congênitos (DCC) A avaliação detalhada do arco aórtico (incluindo sua lateralidade e padrão de ramificação) é crucial durante os exames diagnósticos de DCC, já que ela pode influenciar a incisão cirúrgica ou o bypass cardiopulmonar.^1^ A artéria subclávia aberrante (ASA) ou artéria lusória é uma anomalia do arco aórtico comum. Ela pode originar do arco aórtico à esquerda (AAE) ou do arco aórtico à direita (AAD). A artéria subclávia direita aberrante (ASDA) é a anomalia do AAE mais comum (prevalência de 0,5% a 2%). Mais de 20 configurações de arco aórtico foram descritas. Recentemente foi proposta uma nova classificação para a ASA que distinguiu quatro tipos principais de ASA (com base na lateralidade do arco aórtico e na presença do tronco de carótida comum).^2^ Além disso, a tetralogia de Fallot (ToF) não raramente é acompanhada pelo AAD (até 37% em um estudo por Khan et al.)^3^ Uma artéria subclávia esquerda aberrante (ASEA) com ramificação do AAD também pode estar associada à ToF (21,4% em nosso estudo por tomografia computadorizada cardíaca (TCC) prévio na coorte de ASDA.)^4^ Diferentemente da ASEA, a ocorrência de ASDA (do AAE) em pacientes com ToF até agora só foi relatada casuisticamente. Oswal et al.^5^ identificaram 8 pacientes com ASDA entre 257 de pacientes com ToF.^5^ De Luca et al.^6^ relataram um paciente com ASDA com ToF identificado entre 3334 pacientes (prevalência de ASDA e ToF - 0,03%).^6^ Por último, Nakajima et al.^7^ identificaram sete pacientes com ASDA entre 233 de pacientes com ToF. Entretanto, não foram apresentadas informações sobre se essas ASA se originaram do AAE ou do AAD.^7^ Apresentamos dois pacientes adultos que foram submetidos a correções cirúrgicas de ToF na infância e foram admitidos para avaliação posterior. As modalidades de imagens em ambos revelaram a presença de ASDA com origem no AAE.

### Paciente 1

**Figura 1:**
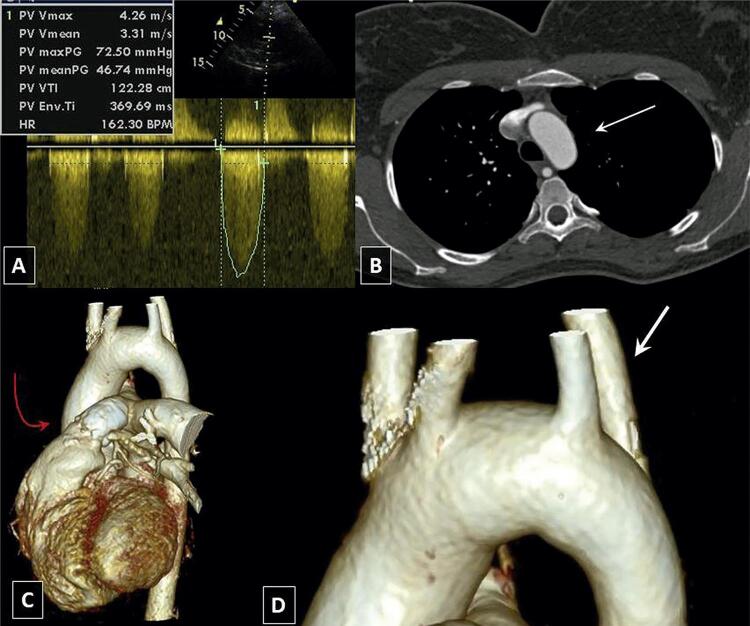
Paciente 1 A) Ecocardiografia transtorácica, vista do eixo curto paraesternal Gradiente de pressão significativo no homoenxerto pulmonar; B) Tomografia computadorizada cardíaca, plano axial. A seta branca indica o arco aórtico à esquerda; C) Tomografia computadorizada cardíaca. Calcificações visíveis do homoenxerto pulmonar no nível da válvula pulmonar (seta vermelha); D) Ampliação do painel “B” com foco na artéria subclávia direita aberrante (seta branca) que se ramifica a partir do arco aórtico à esquerda.

Uma paciente do sexo feminino de vinte e seis anos foi submetida a uma correção de ToF completa aos três anos de idade e permaneceu na classe funcional II de acordo com a New York Heart Association (NYHA). A ecocardiografia transtorácica (TTE) revelou um homoenxerto pulmonar fortemente calcificado com um gradiente de pressão de 72/56 mmHg ( Figura1A ), regurgitação pulmonar (RP) moderada e parede do ventrículo direito (VD) hipertrofiada (12 mm). Fora isso, a função sistólica de ambos os ventrículos estava preservada. A TCC realizada revelou o AAE ( Figura 1B ) com ASDA ( Figuras 1C , D ). Devido à calcificação do homoenxerto, a paciente não se qualificou para o tratamento percutâneo de homoenxerto estenótico e foi tratada de forma conservadora. A TTE realizada 10 anos mais tarde não mostrou nenhum aumento no gradiente de pressão do homoenxerto. O teste de esforço cardiopulmonar (TECP) mostrou diminuição da captação de oxigênio respiratória: 15,3 ml/kg/min (43% do valor previsto) após 2 anos, e 16,9 ml/min/kg (36% do valor previsto) 10 anos mais tarde.

### Paciente 2

**Figura 2:**
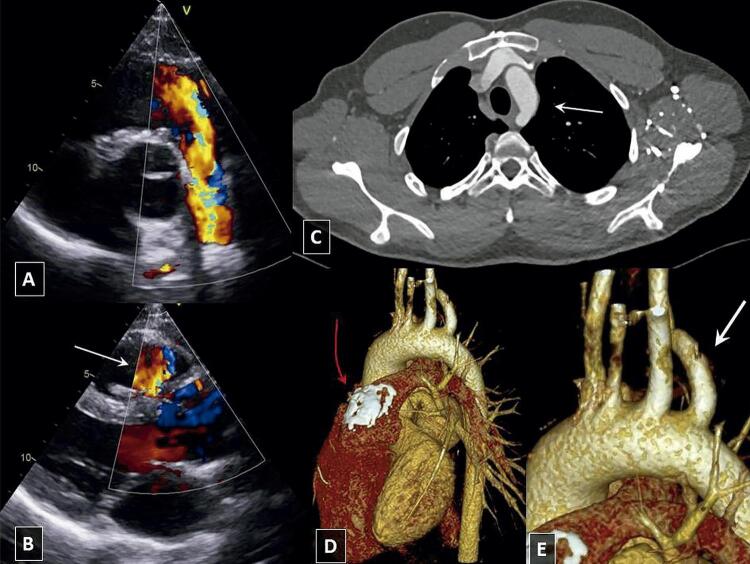
Paciente 2 A) Ecocardiografia transtorácica, vista do eixo curto paraesternal Regurgitação significativa no homoenxerto pulmonar; B) Ecocardiografia transtorácica, vista do eixo longo paraesternal. Jato da regurgitação pulmonar significativa visível no canal de saída do ventrículo direito (seta branca); C) Tomografia computadorizada cardíaca, plano axial. A seta branca indica o arco aórtico à esquerda; D) Tomografia computadorizada cardíaca. Calcificações visíveis do homoenxerto pulmonar no nível da válvula pulmonar (seta vermelha). E) Ampliação do painel “D” com foco na artéria subclávia direita aberrante (seta branca) que se ramifica a partir do arco aórtico à esquerda.

Um paciente do sexo masculino de vinte anos de idade foi submetido ao shunt de Blalock-Taussig com um ano de idade, com correção de ToF completa aos 3 anos de idade. Entretanto, ele precisou fazer uma nova cirurgia após nove meses devido a um shunt da esquerda para a direita residual significativo através de um defeito do septo ventricular congênito e da substituição do homoenxerto pulmonar unicúspide por um homoenxerto pulmonar bicúspide. Ele continuou classificado como classe II da NYHA. Seu TTE mais recente mostrou uma RP significativa no homoenxerto ( Figuras 2A , B ), e um canal de entrada do ventrículo direito aumentado (54 mm) com função sistólica limítrofe do VD (VD S’ 9 cm/s). A função sistólica do ventrículo esquerdo não dilatado estava preservada. A TCC e a ressonância magnética cardíaca revelaram AAE ( Figura 2C ) com ASDA ( Figuras 2D , E ). Devido à anatomia desfavorável do canal de saída do VD, o paciente não era candidato ao tratamento percutâneo da RP e uma abordagem cirúrgica foi oferecida a ele.

Nem o divertículo de Kommerell nem a compressão esofágica estavam visíveis na TCC desses pacientes.

Nosso relatório acrescenta à literatura muito limitada sobre ASDA em pacientes com ToF e tem pontos muito práticos. Primeiramente, a presença de ASDA pode levar ao diagnóstico errado de ramificações do arco aórtico - especialmente durante cirurgias paliativas de emergência antes de a avaliação detalhada por imagem da ramificação do arco aórtico ser feita. Idhrees et al.^8^ relatou sobre um paciente com ToF com ASDA, em que a artéria carótida comum direita (ACCD) foi indevidamente identificada como artéria subclávia. Como consequência, o shunt de Blalock-Taussig foi realizado utilizando-se a ACCD. Isso levou à perda do fluxo sanguíneo da ACCD para o cérebro e a convulsões hipóxicas.^8^ Em segundo lugar, a ASDA pode causar compressão traqueobrônquica (em aproximadamente 10% dos pacientes). Vários casos de fístula esofágica e artéria subclávia no estabelecimento da ASDA (e o sangramento intenso do trato digestivo superior que se segue) já foram relatados, especialmente após a manipulação traqueal ou esofágica posterior a cirurgias cardíacas.^9^ Por último, a canulação cardíaca via artéria radial direita e a ASDA subsequente podem ser um desafio. Portanto, o conhecimento da ramificação do arco aórtico é crucial para a cirurgia de shunt de Blalock-Taussig em pacientes com ToF (embora seja raramente realizada atualmente) ou outra DCC cianótica, além de ser importante para qualquer operação cardíaca ou extracardíaca que exija a manipulação traqueal ou esofágica.
