# Supplementary material for: Tetralogia de Fallot Associada a Artéria Subclávia Direita Aberrante. Implicações Clínicas
Source: Arq Bras Cardiol. 2022 Jul 13;119(3):485–7. [Article in Portuguese] doi: 10.36660/abc.20210880 (PMC9438538; doi:10.36660/abc.20210880)
Supplement: Translation

# Translation: Tetralogy of Fallot Associated with Aberrant Right Subclavian Artery. Clinical Implications

**Authors:** Maciej Michałowski, Pawel Tyczynski, Magdalena Lipczynska, Anna Wójcik, Piotr Hoffman, Adam Witkowski, Ilona Michałowska

**Affiliations:** 1 Polônia National Institute of Cardiology – Department of Interventional Cardiology and Angiology, Warsaw – Polônia; 2 Polônia National Institute of Cardiology – Department of Congenital Heart Diseases, Warsaw – Polônia; 3 Polônia National Institute of Cardiology – Department of Radiology, Warsaw – Polônia

**Keywords:** Heart Defects Congênital, Tetralogy of Fallot, Aorta, Thoracic, Esophageal, Obstruction, Subclavian, Artery

## Abstract

Since the first description of Tetralogy of Fallot (ToF) in 1671 by Niels Stensen and in 1888 by Étienne-Louis Arthur Fallot, numerous papers have reported on this anomaly, along with its variants and concomitant cardiovascular anomalies. Aberrant right subclavian artery (ARSA) is the most common anomaly of the aortic arch. Different from the left aberrant subclavian artery, occurrence of ARSA in ToF-patients has only casuistically been reported so far. The present study reports on two ToF-patients with ARSA. It is important to note that knowledge of the coexistence of both anomalies has highly practical points during surgical or endovascular corrections of congenital heart defects (including ToF).

## Introduction

Anomalies of the aortic arch may be isolated or may be associated with other congenital heart defects (CHD). A detailed assessment of the aortic arch (including its laterality and branching pattern) is crucial during the diagnostic exams of CHD, as it may influence the surgical incision or cardiopulmonary bypass.^1^ An aberrant subclavian artery (ASA) or arteria lusoria is a common aortic arch anomaly. This may originate from the left-sided aortic arch (LAA) or from the right-sided aortic arch (RAA). An aberrant right subclavian artery (ARSA) is the most common LAA-anomaly (prevalence 0.5%-2%). More than 20 aortic arch configurations have been described. Recently a new classification of ASA has been proposed, which distinguished four main types of ASA (based on the aortic arch laterality and the presence of common carotid trunk).^2^ Next, tetralogy of Fallot (ToF) is not rarely accompanied by RAA (up to 37% in a study by Khan et al.^3^ An aberrant left subclavian artery (ALSA) branching-off from RAA may also be associated with ToF (21.4% in our previous cardiac computed tomography (cCT) study on ALSA-cohort.^4^ Different from ALSA, the occurrence of ARSA (from LAA) in ToF-patients has, to date, been reported only casuistically. Oswal et al.,^5^ identified 8 ARSA patients among 257 ToF patients.^5^ De Luca et al.,^6^ reported one ARSA patient with ToF identified among 3,334 patients (prevalence of ARSA and ToF - 0.03%),^6^ Finally, Nakajima et al.,^7^ identified seven ASA patients among 233 ToF patients. However, no details were given whether these ASA originated from LAA or RAA.^7^ The present study reports on two adult patients who underwent surgical corrections of ToF in their childhood and were admitted for further assessment. Imaging modalities in both of the children revealed the presence of ARSA originating from LAA.

### Patient 1

A twenty-six-year-old female patient underwent complete ToF correction at the age of three and remained in functional class II, according to New York Heart Association (NYHA). Transthoracic echocardiography (TTE) revealed a heavily calcified pulmonary homograft with a pressure gradient of 72/56 mmHg ( Fig.1A ), moderate pulmonary regurgitation (PR), and a hypertrophied wall (12mm) of the right ventricle (RV). Otherwise, the systolic function of both ventricles was preserved. Performed cCT revealed LAA ( Figure 1B ) with ARSA ( Figures 1C , D ). Due to calcification of the homograft, the patient was not qualified for percutaneous treatment of the stenotic homograft and was treated conservatively. TTE performed 10 years later showed no increase in the homograft’s pressure gradient. Repeated cardiopulmonary exercise testing (CPET) showed a decreased respiratory oxygen uptake: 15.3ml/kg/min (43% of the predicted value) after 2 years, and 16.9ml/min/kg (36% of the predicted value) 10 years later.

**Figure 1:** Patient 1 A) Transthoracic echocardiography, parasternal short axis view. Significant pressure gradient across the pulmonary homograft; B) Cardiac computed tomography, axial plane. White arrow indicates left-sided aortic arch; C) Cardiac computed tomography. Visible calcifications of the pulmonary homograft at the level of the pulmonary valve (red arrow); D) Magnification of panel “B” with a focus on the right aberrant subclavian artery (white arrow) branching-off from the left-sided aortic arch.

### Patient 2

A twenty-year-old male patient underwent a Blalock-Taussig shunt at one year of age, with complete ToF correction at the age of 3 years. However, he required a re-operation nine months later for a significant residual left-to-right shunt through the congenital ventricular septal defect and replacement of the unicuspid pulmonary homograft with a bicuspid pulmonary homograft. He remained in NYHA class II. His most current TTE revealed significant PR in the homograft ( Fig.2A , B ), together with an enlarged RV-inflow tract (54mm) with borderline RV systolic function (RV S’ 9cm/s). Systolic function of the non-dilated left ventricle was preserved. Both cCT and cardiac magnetic resonance revealed LAA ( Figure 2C ) with ARSA ( Figures 2D , E ). Due to unfavorable anatomy of the RV-outflow tract, the patient was not a candidate for the percutaneous treatment of the PR and was offered a surgical approach.

**Figure 2:** Patient 2 A) Transthoracic echocardiography, parasternal short axis view. Significant regurgitation across the pulmonary homograft; B) Transthoracic echocardiography, parasternal long axis view. Jet of the significant pulmonary regurgitation visible in the right ventricle outflow tract (white arrow); C) Cardiac computed tomography, axial plane. White arrow indicates left-sided aortic arch; D) Cardiac computed tomography. Visible calcifications of the pulmonary homograft at the level of the pulmonary valve (red arrow). E) Magnification of panel “D” with a focus on the right aberrant subclavian artery (white arrow) branching-off from the left-sided aortic arch.

Neither Kommerell’s diverticulum nor the esophageal compression was visible in the cCT of these patients.

Our report adds to the very limited literature of ARSA among ToF patients and has highly practical points. Firstly, the presence of ARSA may lead to a misdiagnosis of the aortic arch branches, especially during emergency palliative surgeries before detailed imaging evaluation of the aortic arch branching has been performed. Idhrees et al.,^8^ reported on a ToF patient with ARSA, in whom the right common carotid artery (RCCA) was misidentified as the subclavian artery. As a consequence, Blalock-Taussig shunt was performed using RCCA. This resulted in a loss of blood flow from RCCA to the brain and hypoxic seizures.^8^ Secondly, ARSA may cause tracheobronchial compression (in approximately 10%). Multiple case reports of subclavian-esophageal fistulae in the setting of ARSA (and subsequent massive upper digestive tract bleeding) have been previously reported, especially after tracheal or esophageal manipulation following cardiac surgeries.^9^ Finally, heart cannulation via the right radial artery and subsequent ARSA may be challenging. Thus, the knowledge of aortic arch branching is crucial for Blalock-Taussig shunt surgery of ToF patients (although rarely performed nowadays) or other cyanotic CHD, as well as matters for any cardiac or extra-cardiac operations requiring tracheal/esophageal manipulation.
